# Erratum

**DOI:** 10.4269/ajtmh.08-0241err

**Published:** 2020-10

In “Genetic Mapping of the Duffy Binding Protein (DBP) Ligand Domain of *Plasmodium vivax* from Unstable Malaria Region in the Middle East” (https://www.ajtmh.org/content/journals/10.4269/ajtmh.2009.80.112), the first author’s name is misspelled. The correct spelling of the author’s last name is Babaeekhou.

The *Journal* regrets the error.

